# Which Sociodemographic and Pathway to Care Factors Influence the Wait Time for Early Intervention for Psychosis? A Mental Health Electronic Health Records Analysis in South London

**DOI:** 10.1111/eip.70087

**Published:** 2025-10-04

**Authors:** Nikki Wood, Jo Hodgekins, Hitesh Shetty, Eduardo Iacoponi, Brian O'Donoghue, Rob Stewart, Sherifat Oduola

**Affiliations:** ^1^ Norwich Medical School University of East Anglia Norwich UK; ^2^ South London & Maudsley NHS Foundation Trust London UK; ^3^ Department of Psychiatry, School of Medicine University College Dublin Dublin Ireland; ^4^ Department of Psychological Medicine, Institute of Psychiatry Psychology & Neuroscience, King's College London London UK; ^5^ School of Health Sciences University of East Anglia Norwich UK

**Keywords:** early intervention psychosis, pathways to care, sociodemographic factors, treatment delays

## Abstract

**Aim:**

In 2016, the Access and Waiting Time Standard (AWTS) was introduced in England, UK, outlining that people with first‐episode psychosis should receive treatment from an early intervention for psychosis (EIP) service within 2 weeks. We examined sociodemographic, pathways to care (PtC), and clinical factors associated with EIP service wait time.

**Method:**

We collected de‐identified data from a large mental health provider in South London, UK. We included patients referred and accepted to EIP services as inpatient or community contacts between 1 May 2016 and 30 April 2019, providing 3 years of data from the introduction of AWTS. Descriptive statistics and multivariable linear regression were performed.

**Results:**

A total of 1806 patients were identified with a mean age of 30 (SD: 10.7) years, of whom 86.3% (*n* = 1559) accessed community EIP and 13.7% (*n* = 247) accessed inpatient EIP; of these, 26.7% were not seen within 2 weeks. Community EIP patients waited longer adj.β = 2.21 days (95% CI: 2.05–2.37) compared with inpatient EIP patients, and being older was associated with longer wait time. Conversely, a shorter wait time was associated with A&E [adj.β = −0.22 days (95% CI: −0.36, −0.10)] and ‘other’ [adj.β = −0.21 days (95% CI: −0.36, −0.03)] PtC characteristics. White non‐British and South Asian patients had shorter wait times compared with White British patients; however, this difference diminished after adjusting for PtC and clinical factors.

**Conclusions:**

Our findings indicate that individual factors, PtC, and mode of contact influence wait time for EIP services. More than a quarter of patients were not seen within 2 weeks, indicating that targeted support in community EIP services is needed to meet clinical guidelines.

## 1 Introduction

### 1.1 Psychosis and Treatment Delays

Delays in accessing appropriate care and treatment for psychosis have been associated with poor quality of life (Penttilä et al. 2014), poorer remission of symptoms and increased relapse rate (Perkins et al. 2005). Therefore, to improve outcomes, treatment delays should be avoided (Norman et al. 2005). Delays can occur at both an individual and service level. Research has identified several factors at an individual level that may prevent people from seeking support from services, such as poor public knowledge of mental health difficulties preventing recognition and help‐seeking (Jung et al. 2017; Murden et al. 2024), lack of insight into symptoms (Penttilä et al. 2014), concerns about stigma associated with psychosis (Martin et al. 2018) and concerns of hospitalisation (Jansen et al. 2018).

At a service level, some studies have shown service delays contribute to treatment delays for psychosis. For example, (Birchwood et al. 2013) reported that non‐specialist services may contribute to a longer duration of untreated psychosis due to structural issues within mental health services, including under‐recognition of symptoms in non‐psychosis services. Oduola et al. (2023) showed that a previous history of mental health service use for non‐psychotic illness is associated with delays in accessing psychosis services. However, it can be difficult to engage patients with psychosis and keep them engaged in treatment (Doyle et al. 2014).

### 1.2 Improving Wait Time to Early Intervention in Psychosis (EIP) services

Many high‐and‐middle income countries have introduced early intervention in psychosis services (Chen et al. 2015; Craig et al. 2004; Henry et al. 2010; Lyne et al. 2015). The central aims of these services include early recognition of symptoms (including identifying at‐risk individuals), reduced delays to treatment, and improved outcomes through clinical and psychosocial interventions (O'Connell et al. 2021; Singh et al. 2018). Support and treatment from early intervention services have been found to improve clinical outcomes, such as reduction in symptoms and relapse rates (Bird et al. 2010), and socioeconomic outcomes, such as employment and housing status (Tsiachristas et al. 2016). Despite these positive outcomes, access to EIP services is unequal and delays still exist. To begin with, the criteria for accessing EIP services can be restrictive; for example, in many contexts, EIP is offered to people under the age of 35 years (O'Donoghue et al. 2024; Oluwoye et al. 2018). Individual factors such as ethnicity (Halvorsrud et al. 2018), educational qualifications (Skrobinska et al. 2024), clinical factors such as DUP (Bhui et al. 2014), and system factors like referral source and help‐seeking (Bhui et al. 2014; Skrobinska et al. 2024) have been linked with variations in pathways and delays to EIP service.

In England (UK), in April 2016, the Access and Waiting Time Standards (AWTS) were implemented in the National Health Service (NHS) designed to (a) reduce waiting times (i.e., at least half of the people referred to EIP services must be offered support or treatment within 2 weeks of referral), (b) extend the age of acceptance for an EIP service from 14–35 years to 14–65 years (NHS England 2016). The two‐week target includes weekends and bank holidays. The clock starts once a central triage point or EIP service receives the referral. The EIP service then assesses whether the person has a first‐episode psychosis (FEP). The assessment of psychosis within EIP services typically includes a physical examination, a complete psychiatric and medical history (Preda and Blackman 2024)). Other areas of assessment include psychological, social, occupational and educational circumstances (NICE 2015). If FEP is confirmed, the clock is stopped following acceptance onto an EIP caseload and a care coordinator has been allocated and engaged with the person. The clock continues if appointments are cancelled or people do not attend (NHS England 2016).

### 1.3 Current Study

Some studies have examined factors associated with wait time to EIP, but most have used data that predates the introduction of AWTS (Kirkbride et al. 2017; Oduola et al. 2023). Additionally, studies that have evaluated the implementation of policy have largely focused on estimating the proportion of people seen within 2 weeks (Adamson et al. 2018; Kreutzberg 2018; Singh et al. 2018). There have been limited studies comprehensively examining wait time for EIP according to sociodemographic, pathways to care (PtC), and clinical factors since the implementation of the AWTS. More evidence of the impact of AWTS on wait time for EIP and the influence of sociodemographic, pathways to care (PtC), and clinical factors are urgently needed to evaluate the policy's effectiveness for diverse populations and identify unmet needs. A recent pre‐AWTS study by (Oduola et al. 2023) examined delays to EIP and the associated individual and clinical factors; however, their sample was identified over 10 years ago, and patients aged ≤ 35 years old eligible for EIS were included. Expanding on Oduola and colleagues' work, this study aimed to provide contemporary insights into factors influencing wait time for EIP using post‐AWTS data. While our study is data‐driven, we expected to observe a reduction in waiting time for EIP and improvement in care pathways in the post‐AWTS era. We addressed the following research questions: (1) What is the median wait time for EIP services, and what proportion of people referred are seen within 2 weeks? (2) What are the characteristics of patients accessing EIP services (inpatient vs. community)? (3) Which sociodemographic, PtC, and clinical factors are associated with waiting times for EIP services? (4) Are there ethnic variations in waiting times for EIP services?

## 2 Materials and Methods

### 2.1 Study Design and Settings

Data for this study were drawn from the South London and Maudsley (SLaM) NHS Trust de‐identified electronic health records. SLaM serves a population of 1.3 million residents in the south London boroughs of Croydon, Lewisham, Lambeth, and Southwark (ONS 2011), with a caseload of around 45 000 people in contact with services at any time. This includes the provision of an EIP service within each borough and (at the time of this study) an inpatient EIP ward based at Lambeth Hospital providing inpatient care for FEP patients across all boroughs (Fusar‐Poli et al. 2020). In 2007, the Clinical Records Interactive Search (CRIS) system was developed which de‐identifies SLaM electronic clinical records for research purposes (Fernandes et al. 2013; Perera et al. 2016; Stewart et al. 2009). Each patient record is de‐identified, coupled with a broader security model to ensure anonymity (see Fernandes et al. 2013 for an in‐depth overview).

Data is available from the CRIS system in two formats: (a) structured fields (e.g., demographic, diagnosis information) and (b) unstructured fields (i.e., free text). We searched the CRIS system for demographic and clinical information using Structured Query Language (SQL) and Natural Language Processing (NLP) to extract data from structured and unstructured fields of the database. Where data is missing, we used the CRIS ‘Front End’ interface (a web‐based searchable interface) to retrieve data manually from each patient record (Perera et al. 2016).

### 2.2 Participants

Data were drawn from the CRIS system using SQL (Perera et al. 2016) based on study inclusion and exclusion criteria. Our sample consisted of patients referred and accepted to the EIP caseload between 1 May 2016 and 30 April 2019, providing 3 years of data from the introduction of AWTS before the Coronavirus pandemic.

### 2.3 Inclusion and Exclusion Criteria

Participants were included if they (a) lived in the London boroughs (Lambeth, Croydon, Lewisham and Southwark) served by SLaM, (b) were accepted to EIP caseload between May 2016 and April 2019, (c) were aged 14–65 years, (d) were presenting with and clinically assessed as having a psychotic disorder or FEP (International Classification of Diseases, Tenth Revision [ICD‐10] codes F20–29) (WHO 1993). Patients were excluded (a) if there was evidence that psychotic symptoms were due to an organic cause or acute intoxication, or (b) if they were aged over 65.

### 2.4 Ethics

CRIS was granted ethical approval by the Oxfordshire Research Ethics Committee (reference 23/SC/0257) as a secondary dataset for research, and we obtained SLaM/CRIS Oversight Committee approval for this study (reference: 22‐032). Under UK law, patient consent was not required for this study.

### 2.5 Procedure and Variables

#### 2.5.1 Sociodemographic Characteristics

Sociodemographic data, including ethnicity, occupation, age, sex, relationship status, education level and employment status, was extracted from CRIS‐structured fields.

Ethnicity is recorded as self‐ascribed by patients in SLaM and based on the 18 categories stated by the UK 2011 census (ONS 2011). For statistical analysis and due to the small numbers in some ethnic groups, we re‐categorised ethnicity as follows: White British, White non‐British (White Irish, Traveller, Other White), Mixed (all mixed ethnic groups), Other (Arab, Chinese, Other), Black African, Black Caribbean, Black Other, South Asian (Indian, Pakistani, Bangladeshi). This is in keeping with previous research in this area (Oduola et al. 2021).

Occupation was extracted using NLP and categorised using the Extended Standard Occupational Classification 2020 Framework (ONS 2020) and collapsed to account for groups that had small numbers. They were categorised as: management/professional, admin, skilled trades, care/leisure, customer services, machine operatives, elementary occupations, student and economically inactive.

Demographic data were operationalised using the Medical Research Council Sociodemographic Schedule (Mallett 1997), in keeping with previous CRIS studies (Oduola et al. 2021, 2023) as follows: age, sex as assigned at birth (male, female), relationship status (single, married/steady relationship, divorced/widowed), education level (no school qualifications, school qualifications, vocational/tertiary qualifications, university qualifications), employment status (unemployed, student, employed).

#### 2.5.2 Sociodemographic Variables With Missing Data

There were some variables with missing data—namely ethnicity, relationship status, employment status, education level and occupation. Missing data were searched and extracted using the CRIS Front End. We interrogated the free‐text fields using the following search terms: ethnicity (white, black, Asian, mixed, language), relationship status (wife, husband, separated, partner, relationship, divorced), employment status and occupation (work, unemployed, job, part‐time, self‐employed, student) and education level (school, college, university, degree, qualification). Two researchers (NW and SO) independently extracted data on missing ethnicity, and an interrater reliability test was performed between the two researchers on a random 10% of the missing sample (*n* = 13). An agreement of 92.3% and a kappa score of 0.90, *p* < 0.001, were achieved, indicating a substantial agreement.

#### 2.5.3 Pathway to Care and Clinical Factors

Wait time, referral source, mode of contact and primary diagnosis were extracted from the structure fields in CRIS, guided by the Personal and Psychiatric History Schedule (WHO 1993).

#### 2.5.4 Wait Time

Wait time was calculated as the date of acceptance by an EIP service minus the date of referral to an EIP service and reported in days. For inpatient EIP, the wait time was the date of admission minus the date of referral, also reported in days.

#### 2.5.5 Pathway to Care Factors

The referral source was determined from the CRIS structured field and coded as a general practitioner (GP), health and social care, accident and emergency (A&E), police/criminal justice agency, self/family, voluntary service or other (i.e., any other source of referral). The mode of contact was categorised based on whether patients had accessed an EIP service through the community EIP or inpatient EIP service.

#### 2.5.6 Clinical Factors

Primary diagnosis was obtained from the CRIS‐structured fields categorised according to ICD‐10 diagnoses (WHO 1993). We organised diagnoses as follows: schizophrenia, acute, schizoaffective disorder, unspecified psychotic disorder, diagnosis not stated.

### 2.6 Statistical Analysis

The data were analysed using STATA 15.1 (StataCorp 2017). Descriptive statistics were used to describe the sample, including frequencies and percentages for categorical data and means, medians, standard deviation, range and interquartile range for continuous data.

To address research question 1, Kruskal–Wallis tests and descriptive percentages were used to estimate the median wait time and the proportion of patients seen within 2 weeks or not. For research question 2, chi‐square tests (and *t*‐test as appropriate) were used to explore the difference between mode of contact (community vs. inpatient) and study variables.

For research question 3, we undertook two sets of analyses. First, Kruskal–Wallis tests were used to analyse the differences between wait time and study variables. Second, we fitted multivariable linear regression analysis using complete data to estimate the associations between wait time and the statistically significant variables in the Kruskal–Wallis. Since the wait time variable was positively skewed, we performed a log transformation to allow for parametric analyses. An arbitrary value of 1 was added to EIP wait time to avoid omitting patients with an inpatient admission or zero values. This approach has been adopted in previous studies (Kirkbride et al. 2017).

For research question 4, we fitted three linear regression models to assess the associations between ethnicity and wait time while controlling for confounders, using the White British group as the reference group. First, we estimated the crude beta coefficients; second, we adjusted for age and sex as a priori confounders. In the third model, we added variables that we associated with wait time in the Kruskal–Wallis test as potential confounders (i.e., referral source, diagnosis and mode of contact).

#### 2.6.1 Sensitivity Analysis

We performed two sensitivity analyses: (a) we dichotomised wait time into ≤ 2 weeks vs. > 2 weeks and examined differences by study variables, and (b) we restricted the sample to the patients with community EIP contact only (see Supporting Information) to assess associations between wait time and sociodemographic, PtC, clinical factors and ethnicity, since the data was skewed by inpatient admission.

Apart from the descriptive statistics reported in Tables 1 and 2, Table S1, all other analyses were conducted with complete data.

**TABLE 1:** Sample characteristics.

Characteristics	*N*	%	M (SD), Mdn (R, IQR)
Age in years (M, SD)			30 (10.17)
EIP wait time (days) (Mdn, IQR)			9 (0–1195, 1–15)
Sex			
Male	1078	59.7	
Female	728	40.3	
Ethnicity^a^			
White British	345	19.1	
White non‐British	168	9.3	
Mixed	96	5.3	
South Asian	115	6.4	
Black African	312	17.3	
Black Caribbean	124	6.9	
Black British	448	24.8	
Other	166	9.2	
Relationship status^b^			
Single	1441	79.8	
Married/Steady relationship	214	11.9	
Divorced/widowed	94	5.2	
Education level^c^			
No school qualifications	81	4.5	
School qualifications	237	13.1	
Vocational/tertiary qualification	408	22.6	
University qualification	820	45.4	
Employment status^d^			
Unemployed	376	20.8	
Student	495	27.4	
Employed	928	51.4	
Occupation^e^			
Management/professional	233	12.9	
Admin	104	5.8	
Skilled trades	87	4.8	
Care/leisure	170	9.4	
Customer services	89	4.9	
Machine operatives	25	1.4	
Elementary occupations	115	6.4	
Student	505	28.0	
Economically inactive	376	20.8	
Referral source^f^			
GP referral	375	20.8	
Health and social care	243	13.5	
A&E referral	609	33.7	
Police/CJA	175	9.7	
Other	334	18.5	
Self/carer	38	2.1	
Voluntary sector	16	0.9	
Primary diagnosis			
Schizophrenia	208	11.5	
Acute	176	9.8	
Schizoaffective disorder	49	2.7	
Unspecified psychotic disorder	697	38.6	
Diagnosis not stated	676	37.4	
Mode of contact			
Community EIP	1559	86.3	
Inpatient ward	247	13.7	

*Note:* Missing data.

Abbreviations: CJA, criminal justice agency; EIP, early intervention in psychosis; IQR, interquartile range; Mdn, median; R, range; SD, standard deviation.

^a^ 32 patients.

^b^ 57patients.

^c^ 260 patients.

^d^ 7 patients.

^e^ 102 patients.

^f^ 16 patients.

**TABLE 2:** Comparisons between community and inpatient EIP services by sociodemographic, pathways to care and clinical characteristics.

Characteristics	*N*	Community EIP *n* = 1559 (%)	Inpatient EIP *n* = 247 (%)	Statistic	df	*p*
Mean age (SD) years		29.49 (10.6)	26.76 (6.1)	*F* = 29.17	1	0.001
Sex						
Male	1078	950 (60.9)	128 (51.8)	*χ* ^*2*^ = 7.36	1	0.007
Female	728	609 (39.1)	119 (48.2)			
Ethnicity						
White British	345	301(19.7)	44 (18.0)	*χ* ^*2*^ = 33.04	7	0.001
White non‐British	168	127 (8.3)	41 (16.7)			
Mixed	96	80 (5.2)	16 (6.5)			
South Asian	115	91 (6.0)	24 (9.8)			
Black African	312	279 (18.3)	33 (13.5)			
Black Caribbean	124	106 (6.9)	18 (7.4)			
Black British	448	389 (25.4)	59 (24.1)			
Other	116	156 (10.2)	10 (4.1)			
Relationship status						
Single	1441	1231 (81.2)	210 (90.5)	*χ* ^*2*^ = 13.16	2	0.001
Married/steady relationship	214	196 (12.9)	18 (7.8)			
Divorced/widowed	94	90 (5.9)	≤ 10 (1.7)			
Education level						
No school qualifications	81	71 (5.4)	10 (4.4)	*χ* ^*2*^ = 1.18	3	0.757
School qualifications	237	204 (15.5)	33 (14.6)			
Vocational/tertiary qualification	408	352 (26.7)	56 (24.8)			
University qualification	820	693 (52.5)	127 (56.2)			
Employment status						
Unemployed	376	332 (21.4)	44 (17.8)	*χ* ^*2*^ = 1.72	2	0.424
Student	495	426 (27.5)	69 (27.9)			
Employed	928	794 (51.2)	134 (54.3)			
Occupation						
Management/professional	233	199 (13.5)	34 (14.5)	*χ* ^*2*^ = 11.80	8	0.160
Admin	104	81 (5.5)	23 (9.8)			
Skilled trades	87	81 (5.5)	12 (2.6)			
Care/leisure	170	152 (10.3)	18 (7.7)			
Customer services	89	77 (5.2)	12 (5.1)			
Machine operatives	25	21 (1.4)	11 (1.7)			
Elementary occupations	115	99 (6.7)	16 (6.8)			
Student	505	432 (29.4)	73 (31.2)			
Economically inactive	376	328 (22.3)	48 (20.5)			
Referral source						
GP referral	375	350 (22.6)	25 (10.3)	*χ* ^*2*^ = 68.29	6	0.001
Health and social care	243	223 (14.4)	20 (8.2)			
A&E referral	609	477 (30.8)	132 (54.3)			
Police/CJA	175	142 (9.2)	33 (13.6)			
Other	334	307 (19.8)	27 (11.1)			
Self/carer	38	34 (2.2)	≤ 10 (1.7)			
Voluntary sector	16	14 (0.9)	≤ 10 (0.8)			
Primary diagnosis						
Schizophrenia	208	184 (11.8)	24 (9.7)	*χ* ^*2*^ = 35.91	4	0.001
Acute	176	157 (10.1)	19 (7.7)			
Schizoaffective disorder	49	36 (2.3)	13 (5.3)			
Unspecified psychotic disorder	697	567 (36.4)	130 (52.6)			
Diagnosis not stated	676	615 (49.5)	61 (24.7)			

Abbreviations: CJA, criminal justice agency; df, degrees of freedom; EIP, early intervention in psychosis; SD, standard deviation.

## 3 Results

### 3.1 Sample Characteristics

A total of 1806 participants were included in the analysis, of whom 1759 had complete data. Table 1 shows that the patients were mostly men (*n* = 1078, 59.7%), Black British, single, university‐educated and students. The largest number of referrals were made by A&E (*n* = 609, 33.7%), followed by GP (*n* = 375, 20.8%) and a high proportion of patients were diagnosed with unspecified psychotic disorder (*n* = 697, 38.6%) or ‘diagnosis not stated’ (*n* = 676, 37.4%).

### 3.2 Wait Time for Accessing EIP Services

The majority of patients contacted community EIP services (*n* = 1559, 86.3%). The median wait time for an EIP service was 9 days (IQR = 1–15). However, when the wait time was dichotomised into ≤ 2 vs. > 2 weeks, we observed that 26.7% of patients were not seen within 2 weeks of referral (see Table S1).

### 3.3 Characteristics of Patients Accessing EIP Services (Inpatient vs. Community)

The largest proportion of patients' mode of contact was via the community (86.3%), compared with via inpatient admission (13.7%). We found strong evidence that the mode of contact (i.e., community vs. inpatient) differed by age, sex, ethnicity, relationship status, referral source and primary diagnosis (see Table 2). Chi‐squared tests showed that patients seen in community EIP were more likely to wait > 2 weeks compared with those admitted to inpatient EIP services (see Table S1).

### 3.4 Wait Time for EIP by Sociodemographic, Pathways to Care and Clinical Factors

Table 3 shows the comparison of wait time by the study variables. Kruskal‐Wallis test revealed differences in wait time and sociodemographic, PtC and clinical factors. In particular, the median wait time was longer for patients aged 35–65 years, by ethnicity (belonging to ‘other’ ethnic group), source of referral (via the voluntary sector, GP or health/social care agency), and diagnosis (i.e., schizophrenia or ‘not stated’). Conversely, the median wait time was shorter for patients accessing inpatient EIP with a diagnosis of schizoaffective psychotic disorder, referred via A&E, Criminal Justice Agency (CJA), of white non‐British ethnic group, and aged 14–35 years old.

**TABLE 3:** Differences in EIP wait time by sociodemographic, pathways to care and clinical factors.

Characteristics	Median EIP wait time in days (IQR)	Kruskal–Wallis test	df	*p*
Age band				
14–35	8 (0–15)	21.948	1	0.001
36–65	11 (6–18)			
Sex				
Male	9 (1–16)	0.716	1	0.398
Female	8 (0–15)			
Ethnicity				
White British	10 (1–21)	24.295	7	0.001
White non‐British	7 (0–14)			
Mixed	9 (0–16.5)			
South Asian	8 (0–14)			
Black African	8 (3–15)			
Black Caribbean	8 (0–15)			
Black British	8 (0–14)			
Other	11.5 (4–20)			
Relationship status				
Single	8 (0–16)	3.714	2	0.156
Married/steady relationship	10 (5–14)			
Divorced/widowed	11 (3–17)			
Education level				
No school qualifications	11 (3–24)	3.287	3	0.349
School qualifications	9 (0–14)			
Vocational/tertiary qualification	8.5 (0–15)			
University qualification	8 (0–15)			
Employment status				
Unemployed	10 (1–17)	3.549	2	0.170
Student	8 (0–16)			
Employed	9 (1–15)			
Occupation				
Management/professional	9 (1–14)	12.479	8	0.131
Admin	7 (0–14.5)			
Skilled trades	8 (2–15)			
Care/leisure	12 (3–20)			
Customer services	9 (0–15)			
Machine operatives	7 (0–14)			
Elementary occupations	7 (0–14)			
Student	8 (0–16)			
Economically inactive	10 (1.5–15)			
Referral source				
GP referral	11 (3–24)	54.503	6	0.001
Health and social care	11 (2–23)			
A&E referral	7 (0–13)			
Police/CJA	8 (0–14)			
Other	9 (2–14)			
Self/carer	10 (2–17)			
Voluntary sector	12.5 (3.5–24)			
Primary diagnosis				
Schizophrenia	10 (1–22)	21.315	4	0.001
Acute	9.5 (2.5–14)			
Schizoaffective disorder	4 (0–14)			
Unspecified psychotic disorder	8 (0–14)			
Diagnosis not stated	10 (1–20)			
Mode of contact				
Community EIP	11 (5–19)	492.307	1	0.001
Inpatient ward	0 (0–0)			

Abbreviations: CJA, criminal justice agency; df, degrees of freedom; EIP, early intervention in psychosis; IQR, interquartile range.

### 3.5 Multivariable Analysis of Associations Wait Time to EIP, Sociodemographic, Pathways to Care and Clinical Factors

Table 4 shows unadjusted and adjusted multivariable linear regression analysis. The results of the unadjusted regression model were consistent with those observed in the Kruskal–Wallis test (see Table 3). When we adjusted for all variables, the strength of association between age (β = 0.01, 95% CI [0.00, 0.01]), source of referral: A&E (β = −0.22, 95% CI [−0.37, −0.10]), other (β = −0.21, 95% CI [−0.37, −0.04]), community EIP services (β = 2.21, 95% CI [2.05, 2.37]) and wait time remained. However, the strength of association for schizoaffective disorder (β = −0.15, 95% CI [−0.50, 0.20]) and unspecified psychotic disorder (β = −0.03, 95% CI [−0.21, 0.14]) diminished (see Table 4). In the sensitivity analysis (see Table S2), in which we included only community EIP data, the strength of association for age and source of referral (i.e., A&E and ‘other’) was held.

**TABLE 4:** Unadjusted and adjusted linear regression of associations between EIP wait time sociodemographic, pathways to care and clinical factors.

	β (95% CI): Unadjusted Models	β (95% CI): Adjusted Model
Age	0.02 (0.01, 0.02)***	0.01 (0.00, 0.01)**
Sex (Female)	−0.06 (−0.19, 0.71)	0.03 (−0.08, 0.14)
Ethnicity		
White British	Reference	Reference
White non‐British	−0.40 (−0.65, −0.15)**	−0.12 (−0.33, 0.10)
Mixed	−0.15 (−0.46, 0.16)	−0.02 (−0.30, 0.23)
South Asian	−0.36 (−0.65, −0.07)**	−0.14 (−0.38, 0.10)
Black African	−0.06 (−0.27, 0.15)	−0.09 (−0.26, 0.10)
Black Caribbean	−0.21 (−0.49, 0.07)	−0.15 (−0.38, 0.10)
Black British	−0.21 (−0.40, −0.02)*	−0.15 (−0.30, 0.01)
Other	0.17 (−0.10, 0.42)	0.02 (−0.18, 0.23)
Referral source		
GP	Reference	Reference
Health and social care	−0.04 (−0.27, 0.18)	0.01 (−0.17, 0.19)
A&E	−0.57 (−0.74, −0.39)***	−0.22 (−0.36, −0.10)**
Police/CJA	−0.44 (−0.68, −0.19)***	−0.13 (−0.34, 0.09)
Other	−0.23 (−0.43, −0.03)*	−0.21 (−0.36, −0.03)**
Self/carer	−0.12 (−0.57, 0.33)	−0.03 (−0.40, 0.35)
Voluntary sector	−0.10 (−0.79, 0.60)	−0.01 (−0.59, 0.56)
Mode of contact (community)	2.28 (2.43, 2.13)***	2.21 (2.05, 2.37)***
Primary diagnosis		
Schizophrenia	Reference	Reference
Acute	−0.04 (−0.32, 0.23)	−0.01 (−0.23, 0.22)
Schizoaffective disorder	−0.52 (−0.95, −0.10)*	−0.15 (−0.50, 0.20)
Unspecified psychotic disorder	−0.21 (−0.42, 0.00)*	−0.03 (−0.21, 0.14)
Diagnosis not stated	0.10 (−0.17, 0.26)	0.09 (−0.17, 0.18)

*Note:* Adjusted Model: all outcomes were adjusted for all the variables in the table.

Abbreviations: CI, confidence intervals; EIP, early intervention in psychosis.

* *p* ≤ 0.05.

** *p* ≤ 0.01.

*** *p* ≤ 0.001.

### 3.6 Ethnicity and Wait Time for EIP Services

In the unadjusted analysis, we observed that patients who were White non‐British (β = −0.40, 95% CI [−0.65, −0.15]), South Asian (β = −0.36, 95% CI [−0.65, −0.07]), and Black British (β = −0.21, 95% CI [−0.40, −0.02]) backgrounds had shorter wait times compared with White British patients (Model 1, Table 5). When accounting for age and sex (Model 2, Table 5), strong evidence remained that White non‐British patients and South Asian patients had shorter wait times for EIP services, but the strength of association no longer held for the Black British patients. Finally, in Model 3, when we added referral source, diagnosis, and mode of contact, the strength of the association of ethnicity with wait time was attenuated (Table 5).

**TABLE 5:** Unadjusted and adjusted linear regression a of associations between ethnicity and EIP wait time (*n* = 1759).

Ethnicity	β (95% CI): Model 1	β (95% CI): Model 2	β (95% CI): Model 3
White British	Reference	Reference	Reference
White non‐British	−0.40 (−0.65, −0.15)**	−0.38 (−0.63, −0.13)**	−0.12 (−0.33, 0.10)
Mixed	−0.15 (−0.46, 0.16)	−0.10 (−0.40, 0.21)	−0.02 (−0.30, 0.23)
South Asian	−0.36 (−0.65, −0.07)**	−0.34 (−0.63, −0.10)*	−0.14 (−0.38, 0.10)
Black African	−0.06 (−0.27, 0.15)	−0.05 (−0.25, 0.16)	−0.09 (−0.26, 0.10)
Black Caribbean	−0.21 (−0.49, 0.07)	−0.19 (−0.47, 0.10)	−0.15 (−0.38, 0.10)
Black British	−0.21 (−0.40, −0.02)*	−0.15 (−0.34, 0.04)	−0.15 (−0.30, 0.01)
Other	0.17 (−0.10, 0.42)	0.14 (−0.11, 0.40)	0.02 (−0.18, 0.23)

*Note:* Model 1, unadjusted; Model 2, adjusted for age and sex; Model 3, adjusted for age, sex, referral source, diagnosis and mode of contact.

Abbreviations: CI, confidence Intervals; EIP, early intervention in psychosis.

* *p* ≤ 0.05.

** *p* ≤ 0.01.

In Model 3, we observed a significant difference in the adjusted *R* ^*2*^ *=* 33.7% compared with *R* ^*2*^ = 2.4% in Model 2, meaning that the source of referral and mode of contact explained most of the variance in the observed associations between wait time and ethnicity. This was confirmed further in our sensitivity analysis (see Table S3), which showed that when we removed inpatient data, there was no difference in EIP wait time for White non‐British, Black British and South Asian ethnic groups after accounting for confounders.

## 4 Discussion

### 4.1 Main Findings

This study examined the sociodemographic pathways to care and clinical characteristics associated with wait time for EIP services. Our analysis showed that age, source of referral and mode of contact were associated with wait time for EIP services. We found that more than a quarter of our sample were not seen in EIP services within 2 weeks of referral. Our initial analysis indicated that White non‐British and South Asian patients had shorter wait times. However, the difference was largely explained by referral sources, e.g., A&E and mode of contact, e.g., inpatient EIP admission.

### 4.2 Comparison of Findings With Previous Research

#### 4.2.1 Sociodemographic Factors

We found that being older was associated with longer wait times for EIP services. This is in keeping with previous studies. For example, Taylor et al. (2023) found that patients over 35 years old experienced more severe symptoms when they presented to EIP services and required less crisis management than their younger counterparts. Oduola et al. (2023), using a sample of FEP patients before the introduction of AWTS, investigated sociodemographic, pathways to care, and clinical factors associated with delay to EIP; they found that being older than 26 years old was strongly associated with longer delays to EIP. Similarly, a service evaluation completed by (Jagger et al. 2020) indicated that patients over 35 years old had more contact with EIP healthcare professionals, especially care coordinators. Alternatively, at the time of our study, it is possible that EIP services were still adapting to accepting older patients under the AWTS (Adamson et al. 2018).

We found that being single and being female increased the likelihood of accessing inpatient EIP, hence having a shorter wait time. This contrasts with previous findings that men with psychotic disorders are more represented in inpatient services than women. Oduola et al. (2023) also found women experienced longer delays to EIP.

Our finding that Black Caribbean, South Asian, Mixed and White non‐British groups were more represented in the inpatient EIP service chimes with other studies (Halvorsrud et al. 2018; Marshall and Rathbone 2011; Gannon et al. 2024; O'Donoghue et al. 2024). Furthermore, UK studies focusing on White Other ethnic groups, such as people from Eastern European backgrounds, have demonstrated that language barriers (e.g., difficulty describing symptoms in their non‐native language), poorer social support, less understanding of healthcare services, stigma (Radez et al. 2024; Maciagowska and Hanley 2018) and lower education levels could all impact help‐seeking (Radez et al. 2024).

#### 4.2.2 Delays to EIP and Mode of Contact

Compared with the median delay to EIP of 120 (IQR; 15–1668) days by (Oduola et al. 2023), we observed a shorter median delay of 9 (IQR; 1–15) days, suggesting a substantial improvement in wait time for EIS pre and post‐AWTS implementation. This change noted, we found that patients who experienced delay were more likely to access support via the community EIP service. In recent times, the National Clinical Audit of Psychosis (NCAP) data shows that most mental health providers in the UK are seeing at least 60% of FEP patients within 2 weeks (i.e., meeting the AWTS targets) (Royal College of Psychiatrists 2022). The NCAP recommends that EIP services would benefit from increased funding, improved staffing levels, as well as culturally informed policies, training and resources to meet the increasing demands (Royal College of Psychiatrists 2022). International studies evaluating public health programmes aimed at improving access to care for psychosis and reducing delays to treatment have shown mixed results. For example, a recent systematic review of the effectiveness of public health interventions, initiatives and campaigns designed to improve pathways to care for individuals with psychotic disorders showed that interventions targeting multiple populations (general public and non‐healthcare professionals) and those lasting > 12 months were more likely to result in a reduction in the duration of untreated psychosis (Murden et al. 2024), hence likely to improve help‐seeking behaviours. However, the authors reported that interventions impacted DUP differently for different groups of patients (Murden et al. 2024).

#### 4.2.3 PtC Factors

Our findings also highlight that the source of referral (A&E, ‘other’ source of referral) and mode of contact (inpatient) are strongly associated with shorter wait times to EIP, indicating an acute presentation and need for urgent care. This is echoed by Senger et al. (2024) in a Canadian EIP study, which found that the rate of urgent health care use was significantly greater for individuals referred to early intervention services from urgent care services compared with those referred via primary care services. Additionally, (Oduola et al. 2023) reported that family involvement in help‐seeking was associated with a shorter delay to EIP. Other studies have also highlighted factors such as stigma (Martin et al. 2018; Lawrence et al. 2021; Jansen et al. 2018) or accessibility issues (Gopalkrishnan 2018; Maraj et al. 2023; NHS England 2016) associated with mental health help‐seeking.

### 4.3 Methodological Considerations

This study contributes to our understanding of the influence of sociodemographic, PtC, and clinical factors on wait time for EIP services, focusing on the inception periods of AWTS. It provides real‐world insights into how the policy is delivered for a representative sample of patients in EIP services in an inner‐city London catchment area. We utilised a large sample from EIP services serving a diverse population, comparing different demographic groups in similar contexts. Furthermore, the large sample size and use of multivariable and sensitivity analysis enabled the identification of variables with significant impact on wait time whilst controlling for confounders.

There are a number of limitations to be borne in mind when interpreting our findings. First, our data source is routinely collected clinical information by clinicians and not necessarily for research purposes. Hence, the quality and accuracy of the data depend on the quality and breadth of documentation. Second, in some ethnic groups, there were small numbers of patients, e.g., people of Chinese and Arab ethnic backgrounds were included in the ‘Other’ ethnic group. Similarly, we collapsed patients from White non‐British, White Irish, Traveller and Other White ethnic backgrounds into one group: White non‐British. This means that any variations in wait time between subgroups were missed. Whilst we adjusted for several sociodemographic PtC factors, it is possible that unmeasured factors, such as living situation, gender, migrant status, and previous service use for other mental health difficulties, may still confound the data. Our study was also limited by the lack of data on PtC characteristics prior to patients presenting to secondary mental health care. Future research would benefit from measuring and accounting for contacts such as primary services, non‐healthcare professionals, and informal help‐seeking contacts with family and friends. This would provide a more complete picture of the factors associated with help‐seeking for psychosis at an earlier stage. Additionally, although our results showed an improvement in wait time for EIP services, it remains unclear whether this improvement is sustained over time. Future research examining the temporal effects of AWTS is warranted.

### 4.4 Implications of Findings

Our study highlights that whilst many patients accessed EIP services within the recommended 2 weeks, delays remain for patients accessing community‐based EIP services. In the UK, workforce shortages within the NHS make it challenging for EIP services to see all patients within 2 weeks (BMA 2021). Nonetheless, there are approaches to and opportunities for reducing wait time for EIP. First, EIP services should prioritise multi‐agency and collaborative working as recommended by the NHS Long Term Plan (NHS England 2019) and support people with psychosis to access support and treatment within their community. Collaborative work with religious groups, voluntary agencies, and charities would likely result in shared knowledge and alternative referral pathways and reduce delays in treatment. Second, whilst A&E presentations are associated with shorter wait times, public health campaigns could help reduce A&E presentations and referrals. This may include campaigns to improve mental health literacy and the identification of psychosis symptoms in the hope of improving and encouraging help‐seeking behaviour via non‐crisis routes. In SLaM, having a dedicated specialist inpatient service for FEP meant that patients were supported within the EIP framework, including the facilitation of referral to community EIP after discharge, which significantly reduced wait times. However, evidence of public health initiatives, community‐level interventions and campaigns aimed at improving access to mental health support among minority ethnic populations in the UK is lacking. Our recent systematic review identified five studies (no studies from the UK), which showed that community‐level interventions have success in promoting help‐seeking for psychosis among ethnic minority populations (Wood et al. 2025). Additionally, partnerships between mental health services and Black faith communities to co‐produce culturally tailored interventions, which is an essential step towards improving access to services, have been advocated in the UK (Codjoe et al. 2024).

Importantly, the incidence of psychotic disorders is associated with the characteristics of the neighbourhoods and the populations living within the neighbourhoods. Our study catchment areas (i.e., the London boroughs of Croydon, Lambeth, Lewisham and Southwark) have a high proportion of residents from ethnic minority backgrounds and relatively high levels of deprivation compared to England overall (Humphreys et al. 2025; Perera et al. 2016 ). It is known that socially deprived areas or those with a higher proportion of migrants or ethnic minorities will have higher rates of psychosis (Kirkbride 2015; Oduola et al. 2021). Therefore, it is essential that EIP services in these areas are funded accordingly to be able to see individuals in these areas in a timely manner and provide them with the comprehensive care required for first‐episode psychosis. Whilst funding for mental health services has been increasing in the UK (NHS England 2019), it has not kept pace with demand. According to the UK National Audit Office, in 2021/22, mental health services spending accounts for around 8% of the total NHS budget (Gilburt and Mallorie 2024), and several of the services are subject to local commissioning arrangements, which result in variations in the type and level of provision available (Gilburt and Mallorie 2024). Therefore, funding areas on a per‐capita basis further disadvantages these areas, and resources should be allocated according to the level of need (Kirkbride 2015). Further research may benefit from including groups of patients that were under‐represented in our sample and by including characteristics such as rural living, area‐level deprivation, living circumstances, socioeconomic status and previous service use to see if this acts as explanatory or modifying variables. In addition, attention to healthcare professionals' cultural awareness, competency, and sensitivity are key to enhancing meaningful engagement with mental health services among minority ethnic people. Gardner and colleagues provide useful recommendations on how mental health professionals can become culturally sensitive to minority ethnic people's needs, including strengthening their access to community resources and peer support, being curious about their culture, and applying cultural and practical adaptations to interventions (Gardner et al. 2024).

## 5 Conclusions

We found that most patients who experienced delays were those seen by community EIP services, and wait time varied according to sociodemographic status, PtC and clinical factors. Our findings suggest patients accessing mental health services via A&E had shorter wait times. However, dedicated resources and better collaboration with the affected populations are needed to realise the benefits of EIP. This could include stronger collaborations with faith leaders and peer support groups, working with educational, non‐profit, and charitable institutions or wider government or healthcare campaigns that could help improve access to care and reduce inequalities in care.

## Conflicts of Interest

R.S. declares research support received within the last 3 years from GSK and Takeda. No other declarations. B.O.D. is an Associate Editor of EIP journal but plays no role in the decision of this manuscript. The other authors declare no conflicts of interest.

## Supporting information


**Table S1:** Comparisons between ≤ 2 weeks vs. > 2 weeks EIP wait time by sociodemographic, pathways to care and clinical characteristics.
**Table S2:** Unadjusted and adjusted linear regression of associations between EIP wait time sociodemographic, pathways to care and clinical factors.
**Table S3:** Unadjusted and adjusted linear regression of associations between ethnicity and EIP wait time, using community EIP data only (*n* = 1517).

## Data Availability

Data sharing is not applicable to this article as no new data were created or analyzed in this study.
