# Supplementary material for: Which Sociodemographic and Pathway to Care Factors Influence the Wait Time for Early Intervention for Psychosis? A Mental Health Electronic Health Records Analysis in South London
Source: Early Interv Psychiatry. 2025 Oct 4;19(10):e70087. doi: 10.1111/eip.70087 (PMC12495505; doi:10.1111/eip.70087)
Supplement: Supplementary file 1 — Table S1: Comparisons between ≤ 2 weeks vs. > 2 weeks EIP wait time by sociodemographic, pathways to care and clinical characteristics. Table S2: Unadjusted and adjusted linear regression of associations between EIP wait time sociodemographic, pathways to care and clinical factors. Table S3: Unadjusted and adjusted linear regression of associations between ethnicity and EIP wait time, using community EIP data only (n = 1517). [file EIP-19-0-s001.docx]

**Title:** Which sociodemographic, clinical, and pathway to care factors influence the wait time for early intervention for psychosis? A mental health electronic health records analysis in South London

**Supplementary Material – Sensitivity Analysis**

**Table S1:** *Comparisons between ≤2weeks vs. >2weeks EIP wait time by sociodemographic, pathways to care and clinical characteristics.*

| Characteristic | ≤2weeks  *N=1,323* (%) | >2weeks  *N=483* (%) | Statistic | *df* | *p* |
| --- | --- | --- | --- | --- | --- |
|  |  |  |  |  |  |
| **Mean age** (SD) | 28.8 (10) | 29.4 (10.2) | *F* = 1.26 | 1 | 0.261 |
| **Sex**  Male  Female | 782 (59.1)  541 (40.9) | 296 (61.3)  187 (38.7) | *X^2^* = 0.70 | 1 | 0.404 |
| **Ethnicity**  White British  White non-British  Mixed  South Asian  Black African  Black Caribbean  Black British  Other | 238 (18.3)  127 (9.8)  69 (5.3)  91 (7)  231 (17.8)  91 (7.0)  341 (26.2)  113 (8.7) | 107 (22.6)  41 (8.7)  27 (5.7)  24 (5.1)  81 (17.1)  33 (7.0)  107 (22.6)  53 (11.2) | *X^2^* = 10.06 | 7 | 0.185 |
| **Relationship Status**  Single  Married/Steady Relationship  Divorced/Widowed | 1051 (82.2)  163 (12.7)  64 (5) | 390 (82.8)  51 (10.8)  30 (6.4) | *X^2^* = 2.44 | 2 | 0.326 |
| **Education Level**  No school Qualifications  School Qualifications  Vocational/tertiary Qualification  University Qualification | 54 (4.7)  178 (15.6)  298 (26.1)  612 (53.6) | 27 (6.7)  59 (14.6)  110 (27.2)  208 (51.5) | *X^2^* = 2.76 | 3 | 0.430 |
| **Employment Status**  Unemployed  Student  Employed | 270 (20.5)  362 (27.5)  685 (52) | 106 (21.9)  133 (27.6)  243 (50.4) | *X^2^* = 0.55 | 2 | 0.759 |
| **Occupation**  Management/Professional  Admin  Skilled Trades  Care/Leisure  Customer Services  Machine Operatives  Elementary Occupations  Student  Economically Inactive  **Referral Source** | 181 (14.5)  78 (6.2)  65 (5.2)  111 (8.9)  65 (5.2)  19 (1.5)  89 (7.1)  369 (29.5)  275 (22) | 52 (11.5)  26 (5.8)  22 (4.9)  59 (13.1)  24 (5.3)  ≤10 (1.3)  26 (5.8)  136 (30.1)  101 (23.4) | *X^2^* = 9.21 | 8 | 0.325 |
| GP referral  Health and Social Care  A&E referral  Police/CJA  Other  Self/Carer  Voluntary Sector | 240 (18.3)  152 (11.6)  496 (37.9)  132 (10.1)  251 (19.2)  28 (2.1)  10 (0.8) | 135 (28.1)  91 (18.9)  113 (23.5)  43 (8.9)  83 (17.3)  10 (2.1)  ≤10 (1.3) | *X^2^* = 53.26 | 6 | 0.001 |
| **Primary Diagnosis**  Schizophrenia  Acute  Schizoaffective Disorder  Unspecified Psychotic Disorder  Diagnosis Not Stated | 136 (10.3)  138 (10.4)  37 (2.8)  551 (41.7)  461 (34.9) | 72 (14.9)  38 (7.9)  12 (2.5)  146 (30.2)  215 (44.5) | *X^2^* = 29.88 | 4 | 0.001 |
| **Mode of Contact**  Community EIP  Inpatient Ward | 1076 (81.3)  247 (18.7) | 483 (100)  0 (0.00) | *X^2^*=104.46 | 1 | 0.001 |
| *N* = number of patients*; df* = degrees of freedom; *SD*= standard deviation;  CJA = Criminal Justice Agency; EIP = Early Intervention in Psychosis | | | | | |

**Table S2:** *Unadjusted and adjusted linear regression of associations between EIP wait time sociodemographic, pathways to care and clinical factors,*

*using community EIP data only (n=1,517***)**

|  | β (95% CI): Unadjusted Model | β (95% CI): Adjusted Model |
| --- | --- | --- |
| **Age** | 0.01 (0.00, 0.01)** | 0.01 (0.00, 0.01)*** |
| **Sex** (Female) | 0.06 (-0.06, 0.19) | 0.04 (-0.09, 0.17) |
| **Ethnicity** |  |  |
| White British | Reference |  |
| White non-British | -0.18 (-0.43, 0.08) | -0.14 (-0.40, 0.01) |
| Mixed | -0.07 (-0.36, 0.23) | -0.25 (-0.32, 0.27) |
| South Asian | -0.21 (-0.49, 0.07) | -0.17 (-0.45, 0.11) |
| Black African | -0.13 (-0.33, 0.07) | -0.10 (-0.30, 0.10) |
| Black Caribbean | -0.19 (-0.46, 0.08) | -0.18 (-0.45, 0.10) |
| Black British | -0.23 (-0.41, -0.05)** | -0.17 (-0.35, 0.01) |
| Other | 0.00 (-0.23, 0.24) | 0.02 (-0.22, 0.25) |
| **Referral Source** |  | |
| GP | Reference |  |
| Health and Social Care | 0.00 (-0.21, 0.21) | 0.01 (-0.19, 0.23) |
| A&E | -0.27 (-0.43, -0.10)** | -0.25 (-0.42, 0.06)** |
| Police/Criminal Justice System | -0.17 (-0.40, 0.01) | -0.14 (-0.37, 0.12) |
| Other | -0.22 (-0.40, -0.03)* | -0.22 (-0.41, -0.02)*** |
| Self/Carer | -0.03 (-0.45, -0.40) | -0.03 (-0.45, 0.40) |
| Voluntary Sector | 0.07 (-0.59, 0.73) | 0.01 (-0.66, 0.67) |
| **Mode of Contact** (community) | Omitted |  |
| **Primary Diagnosis** |  |  |
| Schizophrenia | Reference |  |
| Acute | -0.65 (-0.32, 0.20) | -0.01 (-0.27, 0.25) |
| Schizoaffective | -0.25 (-.68, 0.18) | -0.21 (-0.63, 0.23) |
| Unspecified | -0.07 (-0.28, 0.13) | -0.05 (-0.24, 0.16) |
| Diagnosis Not Stated | -0.01 (-0.21, 0.20) | -0.00 (-0.20, 0.20) |

Adjusted Model: all outcomes were adjusted for all the variables in the table.

**p*≤0.05; ***p*≤0.01; ****p*≤0.001

CI= Confidence Intervals

EIP= Early Intervention in Psychosis

**Table S3**: *Unadjusted and adjusted linear regression of associations between ethnicity and EIP wait time, using community EIP data only (n=1,517)*

| Ethnicity | β (95% CI): Model 1 | β (95% CI): Model 2 | β (95% CI): Model 3 |
| --- | --- | --- | --- |
| White British | Reference |  |  |
| White non-British | -0.18 (-0.43, 0.08) | -0.17 (-0.42, 0.08) | -0.14 (-0.40, 0.01) |
| Mixed | -0.07 (-0.36, 0.23) | -0.05 (-0.34, 0.25) | -0.25 (-0.32, 0.27) |
| South Asian | -0.21 (-0.49, 0.07) | -0.20 (-0.48, 0.08) | -0.17 (-0.45, 0.11) |
| Black African | -0.13 (-0.33, 0.07) | -0.13 (-0.33, 0.07) | -0.10 (-0.30, 0.10) |
| Black Caribbean | -0.19 (-0.46, 0.08) | -0.19 (-0.45, 0.08) | -0.18 (-0.45, 0.10) |
| Black British | -0.23 (-0.41, -0.05)** | -0.20 (-0.38, -0.02)* | -0.17 (-0.35, 0.01) |
| Other | 0.00 (-0.23, 0.24) | -0.00 (-0.23, 0.23) | 0.02 (-0.22, 0.25) |

Model 1 – unadjusted

Model 2 – adjusted for age and sex

Model 3 – adjusted for age, sex, referral source and diagnosis.

**p*≤0.05; ***p*≤0.01; ****p*≤0.001

CI= Confidence Intervals

EIP= Early Intervention in Psychosis
